# Navigating challenging patient factors in systemic therapy for head and neck cancer

**DOI:** 10.1093/oncolo/oyaf035

**Published:** 2025-05-16

**Authors:** Petr Szturz, Robert I Haddad, Marshall Posner, Jan B Vermorken

**Affiliations:** Department of Oncology, University of Lausanne and Lausanne University Hospital, 1011 Lausanne, Switzerland; Department of Medical Oncology, Center for Head & Neck Oncology, Dana-Farber Cancer Institute, Boston, MA 02215, United States; Tampa General Hospital/Cancer Center of South Florida, Tampa and West Palm Beach, FL 33461, United States; Faculty of Medicine and Health Sciences, University of Antwerp, 2610 Wilrijk (Antwerp), Belgium; Department of Medical Oncology, Antwerp University Hospital, 2650 Edegem, Belgium

**Keywords:** head and neck cancer, systemic therapy, contraindications, clinical trials, real-world data, decision-making, efficacy, adverse events, quality of life

## Abstract

Patients with head and neck cancer often present with complex challenges due to a substantial comorbidity burden, including substance use disorders, and the tumor’s location in regions that are both cosmetically and anatomically sensitive. These challenges can be categorized into 6 areas, that is, overall health (eg, performance status, biological age), physiological life stages (eg, aging), organ dysfunctions (including autoimmune comorbidities, organ transplants, and psychiatric disorders), previous and concurrent malignancies, previous and current therapies, and adherence to therapy. We provide a practical guide to help physicians understand and address the phenotypic multitude of potential complications in the management of these patients. The process has 4 main phases involving identification of the clinical challenge, understanding the reasons for ineligibility (contraindications), assessment of the risk to benefit ratio, and finally making informed decisions about systemic treatment. Proactive interventions, including prehabilitation, are crucial for optimizing patient outcomes and reversing some ineligibility issues. The evidence supporting contraindications is drawn from both clinical trials and real-world data, each with its strengths and limitations. These contraindications are applied as absolute or relative and further refined by expert opinions and consensus statements. There are 2 main reasons for ineligibility for a given treatment, absence of supporting data or negative outcome data. In these cases, careful interpretation using all levels of clinical evidence, including extrapolation and preclinical rationale, is essential. By mastering these skills, that may in the future be enhanced by artificial intelligence methods, significant advancements in patient care can be achieved.

**Implications for practice**

Patients with head and neck cancer with challenging characteristics often require individualized approaches to balance effective treatment and toxicity management and may not always fit into rigid and idealized treatment guidelines. This paper offers a framework to guide physician decision-making, emphasizing the importance of understanding the complexity of comorbidities, including substance use disorders as well as the strengths and weaknesses of different data sources ranked by the level of evidence they provide. By gaining insights into the reasons for ineligibility, exploring strategies to reverse or circumvent contraindications, and identifying means to assess and modify recommended therapies for the broader, less idealized patients, treating physicians can optimize their systemic treatment choices.

## Introduction

**Figure 1.**
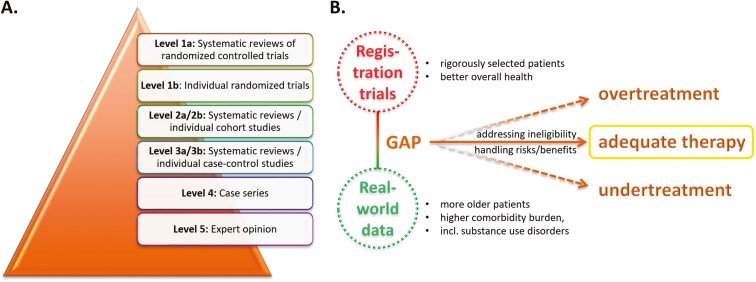
(A) The different levels of evidence align with the recommendations of the Oxford Centre for Evidence-Based Medicine.^1^ (B) The complexity of decision-making in head and neck cancer is underscored by the gap between registration trials and the real-world setting, corresponding to higher and lower levels of evidence, respectively. This gap, depicting the difference between clinical trial participants and the majority of patients seen in daily clinical practice, increases the risk of both overtreatment and undertreatment. An informed use of absolute and relative contraindications, along with careful handling of the risks and benefits (see Question 6), helps guide the selection of appropriate therapies for our patients.

The general approach to clinical decision-making has evolved over the past decades and is currently based on a consensus interpretation of scientific evidence from various sources, reflected in official practice recommendations that are adjusted to individual patients by treating physicians. According to the Oxford Centre for Evidence-Based Medicine, the sources of evidence supporting our decisions can be classified into 5 levels, with the lowest risk for information bias provided by systematic reviews of randomized controlled trials (level 1a), followed by individual randomized trials (1b), then systematic reviews of cohort studies (2a) and individual cohort studies (2b). Analogously, level 3 concerns case-control studies. Level 4 relates to case series and poor-quality cohort and case-control studies, while level 5 is purely based on expert opinion (Figure 1A).^1^

With a natural tendency to prioritize the highest level of evidence, that is, large, randomized trials, and the typically associated requirement to enroll only rigorously selected participants, the gap between such study populations and real-world head and neck cancer (HNC) patients, known for a high burden of comorbidities, has been widening.^2,3^ In clinical practice, the prevalence of comorbid conditions in HNC is about 60%, with one-third classified as severe and even higher rates observed in older adults.^2^ Contrastingly, patients selected to participate in clinical trials generally have better overall health, including performance status.^4^ Approximately 20% of patients with cancer are not eligible for trial inclusion, and, interestingly, around 25% of early terminated HNC trials are closed due to insufficient accrual.^5-7^ However, these numbers should be interpreted in the context that, according to United States estimates, only 7.1% of patients with cancer are enrolled in treatment trials.^8^

This discrepancy generates various pitfalls in managing HNC patients, particularly if they have clinically challenging characteristics. On the one hand, when the absence of clinical trial counterparts is highlighted, real-world patients with challenging features may receive less aggressive treatment, potentially leading to worse efficacy outcomes despite being able to tolerate standard therapy. On the other hand, if similarities with trial populations are emphasized, such patients might undergo standard therapy, resulting in serious complications mediated by their comorbidities (Figure 1B).

We have divided this paper into 7 chapters addressing 7 principle questions pertaining to the management of challenging cases with mucosal HNC. Our objective is to showcase both clinical trials and real-world data as sources for clinical decision-making and implement them in an algorithm guiding the choice of systemic treatment in the presence of formal contraindications.

### Question 1: What are potentially challenging patient factors?

As summarized in Table 1, these factors can be divided into 6 categories, mainly comprising pathological states but also involving physiological life stages that influence patient care throughout life. Depending on their clinical manifestations, the different patient factors represent potential contraindications for systemic therapy. Several scales and tools have been developed to describe them, and the correct diagnosis and assessment of their phenotypic penetrance are fundamental to guide further decision-making.

**Table 1.** Patient-related factors that, depending on the degree of deterioration, may represent contraindications for systemic anticancer therapy.

Baseline characteristics	Specific considerations
Overall health	Performance status
	Biological age
Physiological life stages	Aging
	Reproductive age
	Pregnancy
	Lactation
Organ functions	Cytopenias
	Neurological disorders
	Cardiopathies
	Vasculopathies, thrombosis, bleeding diathesis
	Pneumopathies
	Digestive disorders, including liver dysfunction
	Musculoskeletal disorders
	Endocrinopathies
	Sensory disorders
	Psychiatric disorders
	Autoimmune diseases
	Organ transplants
Previous and concurrent malignancies	Previous malignancies (of the same or a different origin as the index cancer)
	Concurrent malignancies
Previous and current therapies	Previous anticancer and immunosuppressive therapies
	Current medication
	Alternative and complementary medicine
Compliance	Understanding the therapy, communication with healthcare providers
	Comorbidities, substance use disorders
	Social, psychological, and emotional support

In particular, aging is crucial to the management of HNC, owing to the growing number of older people with cancer, both in absolute terms and relative to the general population. These epidemiological trends hold true for HNC, where the majority of cases are now diagnosed in adults 65 years or older, with projections indicating a steady increase in this proportion.^9^ When caring for geriatric patients, the following aspects should be considered. Older adults with HNC have been underrepresented in clinical trials, and trials focusing specifically on this population are extremely rare.^10^ Given the complexity of accompanying health issues in the elderly, often referred to as the biological age, geriatric assessment tools should be employed. Fit older individuals can derive the same benefits from anticancer therapies as their younger counterparts, albeit with a higher risk of adverse events due to the physiological decline in organ reserves.^11^ In the present era marked by the advent of modern immunotherapy and other targeted approaches, along with advances in supportive care, the role of clinical estimates of patients’ performance status has gradually diminished.^12^ Moreover, in older cancer patients, the correlation between Eastern Cooperative Oncology Group (ECOG) performance status and functional status measured by Activities of Daily Living (ADL) and Instrumental Activities of Daily Living (IADL) is only moderate whereas it is absent between performance status and comorbidities, chronological age, and tumor stage.^13^ Older adults over 65 or 70 years of age may thus benefit from geriatric screening tools followed, if necessary, by a comprehensive geriatric assessment.

Even at baseline, organ function can be graded using the Common Terminology Criteria for Adverse Events (CTCAE). Previous malignancies may have been linked to prior treatment with systemic anticancer drugs that may represent potential contraindication for further retreatment, such as when cisplatin, known for a correlation between its cumulative dose and the risk of adverse events, especially nephro-, neuro-, and ototoxicity and potentially gastrointestinal, hepatic, and hematological toxicities, were given for example for prior testicular cancer or when the patient had previously been exposed to taxanes (paclitaxel or docetaxel) associated with neurotoxicity.^14-17^ A longer treatment-free interval can reduce the risk of some cumulative toxicities, but high-level evidence for this assumption is lacking. Of note, cumulative toxicity, which is critical for determining the potential reuse of the same drug later in the disease course, should be distinguished from chronic and late toxicities, which refer to persistent or delayed adverse events, respectively. Interestingly, prior radiotherapy, though not a contraindication per se, can impact tolerance to subsequent systemic treatments. The so-called radiation recall reactions may occur in previously irradiated regions, potentially causing skin, mucosal, or lung damage upon the introduction of chemotherapy (eg, taxanes or capecitabine), targeted therapies (including the epidermal growth factor receptor inhibitors erlotinib and cetuximab), or immune checkpoint inhibitors, even years later.^18,19^

Redefining the therapeutic paradigms in many oncologic diagnoses, immune checkpoint inhibitors were introduced to clinical practice more than a decade ago. While generally very well tolerated, severe and potentially life-threatening immune-related adverse events may reappear upon retreatment, but they can be mitigated with appropriate prophylactic approaches.^20^ Knowledge about prior treatment with immunosuppressive therapies may also reveal an intercurrent autoimmune disease, which, although not active at present, may recur when immune checkpoint inhibitors are initiated, particularly if immunosuppressants have been recently discontinued. The concurrent use of immunosuppression represents a relative contraindication to immune checkpoint inhibitors, more due to a scarcity of supporting data than the risk of unmanageable consequences, though the antitumor response may be lower.^21^ Similarly, some organ transplant recipients may safely receive immunotherapy, even without reducing their immunosuppression.^22^

An important component of each list of current medications is also an overview of complementary, over the counter, drugs taken by almost one-quarter of these patients, which may be responsible for pharmacodynamic interactions.^23^ Finally, adherence to therapy in HNC patients can be compromised by socioeconomic factors (eg, income, education, employment, social support), health-care team and system-related factors (eg, problematic health services or insurance, understanding the therapy, communication with healthcare providers), condition-related factors (ie, disease symptoms and its complex impact on patient well-being), therapy-related factors involving side effects and scheduling, and patient-related factors (eg, comorbidities, psychological and emotional factors, and substance use disorders covered in Question 7).^24^

### Question 2: Why have contraindications been established?

There are 2 main reasons why contraindications have been introduced into clinical practice, that is, to avoid excessive toxicity of systemic anticancer therapy that is being administered and to prevent an unintended decrease in its efficacy. Two additional objectives require attention; the first is to refrain from patient non-compliance with treatment, affecting both toxicity and efficacy, and the second is to forestall impaired data interpretation, which may occur if another concurrent malignancy presents with metastases (lymphogenic or hematogenic) that are indistinguishable from such manifestation of the index cancer on imaging. This issue becomes particularly evident in unresponsive cases and is commonly resolved through obtaining histological verification of equivocal lesions. The most common synchronous primary tumors, apart from head and neck carcinoma, include lung and esophageal cancers but also other digestive cancers and bladder carcinomas.^25^ Reduced adherence to antineoplastic therapy with prolonged interruptions can lead to insufficient exposure, repopulation with resistant cancer cells, and ultimately treatment failure.^26^ In this respect, the timely initiation of curative treatment and the avoidance of prolongation of its overall duration are essential for achieving optimal results, which places additional demands on meticulous pretreatment evaluation to address possible baseline challenges.^27^ The impact of impaired compliance on toxicity is less pronounced but may become evident when healthcare professionals are not available to provide timely management or are unaware of the possibility of immune-related adverse events if the patient consults a different institution.

Excessive toxicity can be directly linked to comorbidities, when they are worsened by the respective drugs (eg, nephrotoxic drugs given in renal insufficiency) or when other side effects are more pronounced in the presence of a given comorbidity (eg, hematotoxic drugs given in renal insufficiency). An indirect effect of comorbidities on excessive toxicity may occur through drug interactions between antineoplastic agents and current medications taken for these comorbidities. Similar consequences may result from the concomitant use of alternative or complementary medicine, although these approaches may not always be undertaken to address comorbidities but also the oncologic diagnosis itself. Special attention should be given to previous exposure to antineoplastic drugs leading to subclinical organ dysfunction, which may progress to overt disease in the case of retreatment, as explained above.

Decreased efficacy is a less common observation than excessive toxicity but can be encountered in the context of specific interactions with both concomitantly prescribed medicines and alternative and complementary approaches. Tables 2 and 3 provide an overview of this topic, involving examples for each of the categories with drugs used in HNC, namely platinum derivates, fluoropyrimidines, taxanes, methotrexate, the epidermal growth factor receptor inhibitor cetuximab, and the immune checkpoint inhibitors nivolumab and pembrolizumab blocking the signaling pathway of programmed cell death protein-1 (PD-1). Reference data are available in the official Summaries of Product Characteristics and in some further publications on chemotherapy and immunotherapy.^14-17,21,28,29^

**Table 2.** Reasons certain systemically administered anticancer drugs may be contraindicated in specific clinical scenarios due to the risk of increased toxicity.

Excessive toxicity					
Mechanism: 1. Direct effect of comorbidities and patient-related factors					
Type	Clinical example			Potential undesired outcomes	
The same toxicity-comorbidity interaction	Nephrotoxic cisplatin in renal insufficiency			Worsening of renal insufficiency	
	Platinum or cetuximab in electrolyte disbalances			Worsening of the electrolyte disbalances	
A different toxicity-comorbidity interaction	Non-nephrotoxic capecitabine in renal insufficiency			Worsening of overall toxicity	
	Paclitaxel in hepatic insufficiency			Worsening of myelotoxicity	
	5-fluorouracil in DPD deficiency			Worsening of overall toxicity	
	Methotrexate in folic acid deficiency			Worsening of overall toxicity	
	Chemotherapy in immunodeficiency disorders (AIDS), malnutrition			Worsening of overall toxicity	
	5-fluorouracil, capecitabine, and supportive drugs (domperidone, ondansetron) in patients with risk factors for QTc prolongation			Ventricular arrythmia (torsade de pointes)	
	Any drug in the presence of pre-existing allergies			Allergic reactions	
	Immune checkpoint inhibitors in organ transplant patients			Transplant reject	
Comorbidities and substance use disorders	Compromised adherence to therapy			Worsening of neglected toxicity	
Socio-economic factors	Compromised adherence to therapy			Worsening of neglected toxicity	

Mechanism: 2. Indirect effect of comorbidities					
Type	Clinical example		Potential undesired outcomes		
Through drug interactions	Cisplatin combined with nephrotoxic or ototoxic drugs		Worsening of nephrotoxicity and ototoxicity		
	Taxanes with CYP3A4 inhibitors (ketoconazole, clarithromycin)		Worsening of overall toxicity		
	5-fluorouracil with folic acid and/or brivudine		Worsening of overall toxicity		
	Methotrexate with NSAIDs, proton pump inhibitors, or antibiotics (penicillins)		Worsening of overall toxicity		
	Methotrexate with hepatotoxic drugs (azatrioprine, antifolates incl. sulfonamides)		Worsening of hepatotoxicity		
	Chemotherapy with immunosuppressive drugs		Increased risk of infections		
Through interactions with alternative and complementary medicines	Taxanes with grapefruit, chamomile, or green tea (inhibits CY3A4)		Worsening of overall toxicity		
Previous exposure to the same anticancer agent	Cisplatin		Increased risk of nephrotoxicity, neurotoxicity, and ototoxicity		
	Taxanes		Increased risk of neurotoxicity		
Previous radiotherapy	Taxanes, capecitabine, cetuximab, immune checkpoint inhibitors		Radiation recall reactions		

Abbreviations: AIDS, Acquired Immune Deficiency Syndrome; DPD, dihydropyrimidine dehydrogenase; NSAIDs; non-steroidal anti-inflammatory drugs.

**Table 3.** Reasons certain systemically administered anticancer drugs may be contraindicated in specific clinical scenarios due to the risk of decreased efficacy.

Decreased efficacy		
Mechanism: 1. Direct effect of comorbidities or patient-related factors		
Type	Clinical example	Undesired outcomes
Comorbidities and substance use disorders	Compromised adherence to therapy	Decreased efficacy
Socio-economic factors	Compromised adherence to therapy	Decreased efficacy

Mechanism: 2. Indirect effect of comorbidities		
TYPE	Clinical example	Undesired outcomes
Through drug interactions	Cisplatin with chelating agents (penicillamin) or pyridoxin	Decreased efficacy
	Immune checkpoint inhibitors with proton pump inhibitors, antibiotics, or glucocorticoids (> 10 mg/day of prednisone equivalent)	Decreased efficacy
Through interactions with alternative and complementary medicines	Taxanes with St. John’s Wort (Hypericum perforatum induces CYP3A4)	Decreased efficacy
	Taxanes with echinacea (induction of CYP3A4)	Decreased efficacy
	Cisplatin with Chinese skullcap (Scutellaria baicalensis)	Less nausea but also decreased efficacy
Previous exposure to the same anticancer agent	Limited possibility of retreatment	Decreased efficacy if there is no equipotent alternative

Abbreviations: DPD, dihydropyrimidine dehydrogenase; NSAIDs; non-steroidal anti-inflammatory drugs.

### Question 3: How is evidence about contraindications generated, applied, and improved?

**Figure 2.**
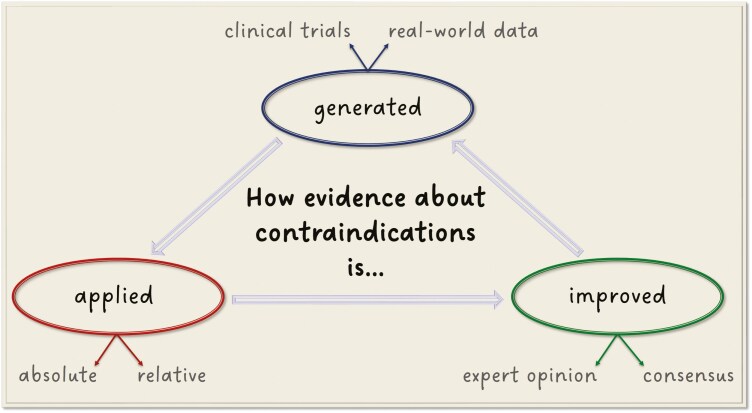
Lifecycle of evidence about contraindications to systemic anticancer drugs.

Figure 2 shows that such evidence can be generated either from prospective clinical trials or real-world data, and it can be applied as either absolute or relative contraindications. The latter category typically relies on real-world data, which allows for the establishment of specific clinical scenarios that may be more challenging to reproduce in a controlled clinical environment. Contraindications are treatment-specific, meaning that source data reproducing a particular clinical situation as closely as possible should be sought, with preference for large randomized trials that provide the highest level of evidence.

Hence, clinical trials outline the framework by defining the permitted and prohibited values of clinical, paraclinical, and demographic parameters. However, these trials may not always sufficiently reflect individual patient characteristics in daily clinical practice. Some patients may still derive significant benefit from the study treatment even if they do not fully comply with the rigid trial criteria, which are sometimes designed more to minimize the overall rate of complications than to address treatment-related adverse events based on the pharmacokinetic and pharmacodynamic properties of the drug. Such restrictive conditions represent absolute contraindications for a given therapeutic approach but may be mitigated by introducing the concept of relative contraindications (see Question 5).

Addressing the gap between clinical trials and real-world data through the use of absolute and relative contraindications helps us select the most appropriate therapy for our patients, preventing both overtreatment and undertreatment. The latter may occur when patients do not qualify for a treatment they could have benefitted from because supporting real-world data has not been consulted. In Question 4, we will explain that treating patients by analogy to clinical trials may loosen some strict criteria but also carries the risk of overtreatment, which can, in turn, again be mitigated by real-world data (Figure 1B).

Finally, the evidence and its application can be further improved through expert opinions, particularly in the frame of consensus statements. Although this inherently represents a form of real-world evidence, it has the unique advantage of incorporating theoretical assumptions and hypotheses that are not necessarily linked to previously treated patients. Therefore, expert opinions can address any clinical situation, even those where outcomes have not yet been described or observed. Despite being a double-edged sword, capable of solving complex clinical dilemmas yet carrying the risk of unpredictable consequences, it is a vital addition to everyday clinical decision-making, enabling the holistic care patients require. Nevertheless, panel opinions, including tumor boards, and consensus statements should be prioritized whenever feasible to minimize personal bias and inter-rater variability.

### Question 4: Do we treat according to clinical trials, by analogy to them, or using real-world data?

Contraindications generated using prospective clinical trials on the one hand, and real-world data on the other, may have different quality and applicability in an individual patient. Registration trials are typically randomized studies with well-defined patient selection criteria which, albeit less strict than in the early phase setting, pose a barrier to about 20% of patients seen in clinical practice.^5,6^ This is also the reason why initiatives have been undertaken to broaden the eligibility criteria to make clinical trials more inclusive and thus more representative and useful in guiding clinical decision-making in routine practice.^30^Table 4 provides an overview of the advantages and disadvantages of using clinical trials and real-world data for decision-making. The former approach can be employed in 2 different ways, either rigorously, when patients must qualify according to the eligibility criteria, primary outcomes, or subgroup analyses of a trial exploring the respective therapy, or more loosely, when some criteria are adjusted to fit individual patients, treating them by analogy to the respective clinical trials. The latter concept stands between clinical trials-based and real-world-based approaches, as it may apply some retrospective observations to amend the trial eligibility criteria.

**Table 4.** Selection of the most appropriate systemic therapy for a given patient can be based on the eligibility criteria of a clinical trial investigating the respective drug or therapeutic approach, by analogy to such criteria, or on real-world data. Each approach has its strengths and limitations, which are summarized in this table.

According to clinical trials		By analogy to clinical trials		Using real-world data	
Pros	Cons	Pros	Cons	Pros	Cons
Higher chances of reproducing positive trial results	May be too restrictive, excluding potential candidates	More inclusive approach	Less expectable efficacy and toxicity	Larger size and scale of datasets	Confounding and selection biases may preclude generalizability
Expectable toxicity	Eligibility criteria cannot be blindly followed, as some trials may have shown worse toxicity and/or efficacy in certain patient subgroups		Legally less secure	More complex patient appraisal	Lack of rigorous inclusion and exclusion criteria
Legally more secure	Subgroup analyses of toxicity outcomes according to different baseline organ functions are often missing			Risk factor analysis	Control groups are not always available
	Physicians, being aware of drug toxicity profiles, may have tended to avoid enrolling patients with borderline baseline functions despite the patients being formally eligible			Different clinical scenarios	Missing data are more common than in clinical trials
	Trial populations have usually a lower overall comorbidity burden than the real-world population hampering reproducibility of the results			Possibility to better explore relative contraindications by focusing on highly selected, albeit potentially small, populations	Difficult investigations of the most recent therapeutic and diagnostic approaches
	Some less frequent but still clinically significant adverse events may not be reported				

Real-world data can be used in 3 scenarios. First, to validate or refute the outcomes of clinical trials, thus answering the question of whether evidence from clinical trials is relevant or not. In head and neck oncology, this approach proved beneficial when assessing the contribution of cetuximab to the therapeutic portfolio. In the locally advanced setting, the initial excitement brought by the results of the IMCL-9815 phase III trial was dispelled by retrospective observations and later by prospective trials, so currently cetuximab, when combined with radiotherapy, should not be preferred to cisplatin but is probably better than radiotherapy alone.^31-34^ On the other hand, the success of the palliative first-line treatment combining cetuximab with a platinum/5-fluorouracil backbone was consistently reproduced in real-world population, and the regimen has remained a valid comparator arm until present, albeit with a more specifically selected population of patients harboring tumors without PD-1 ligand (PD-L1) expression.^35,36^ Second, real-world data can be used to broaden the target population for a given treatment, such as in the presence of relative contraindications or in rare diseases. Lastly, real-world data contribute to the development of best-practice approaches.^37^ Beyond efficacy, additional information about toxicity is provided through pharmacovigilance, ie, post-marketing drug safety surveillance, which may capture previously unreported complications, with findings influenced by the frequency of clinical visits.^38^ While the generation of real-world evidence may be more feasible, attention is required when translating the results to clinical practice as outlined in Table 4.

In daily routine, the preference for one approach over another is not always a matter of choice but sometimes a matter of availability.

### Question 5: What is the difference between absolute and relative contraindications?

In HNC, this question has repeatedly been addressed with respect to cisplatin due to its central role in the management of these patients and its substantial risk of various side effects.^39-41^ Absolute contraindications have usually been established by clinical trials, often using arbitrary cutoff values. They are typically based on eligibility criteria, less frequently on trial outcomes and subgroup analyses. Importantly, despite exploring similar approaches, the definition of eligibility criteria regarding organ functions has not been uniform, particularly concerning the class-specific toxicities of cisplatin, that is, nephrotoxicity, ototoxicity, and neurotoxicity. In different trials, adequate organ functions at baseline have been defined with varying thresholds, such as glomerular filtration rates between 45 and 70 mL/minute, pre-existing hearing loss that is either not clinically significant or not audiometrically assessed to be more than grade 1 or sometimes even 2, and pre-existing neuropathy that is not more than grade 1, rarely 2.^42-50^

Such discrepancies in the description of eligibility criteria in clinical trials provide an opportunity to justify and delineate their relativeness. This approach is particularly feasible when absolute contraindications are defined differently across studies, and the creation of relative criteria represents an effort to address this variability. Contrastingly, certain absolute criteria, like pregnancy, lactation, and hypersensitivity reactions, are not subject to variability across clinical trials but they can be de-escalated to a relative status under certain circumstances on a case-by-case basis, for example, by restricting chemotherapy to the second or third trimesters of pregnancy or performing desensitization in cases of immediate and non-severe delayed reactions.^51,52^ Alternatively, the development of relative criteria may occur when no absolute criteria have been established for a given condition, as is the case with socio-economic status, certain drug interactions, prior exposures to anticancer therapies, weight loss, or biological age determined through geriatric screening. The relativeness of these criteria may arise from their inclusion in only some but not all studies in a given field of interest or from insufficient knowledge about their clinical impact.

Both categories often require interpretation from opinion leaders and expert panels, as well as recommendations from consensus statements (see also Question 3).

### Question 6: How to approach a patient with challenging factors?

**Figure 3.**
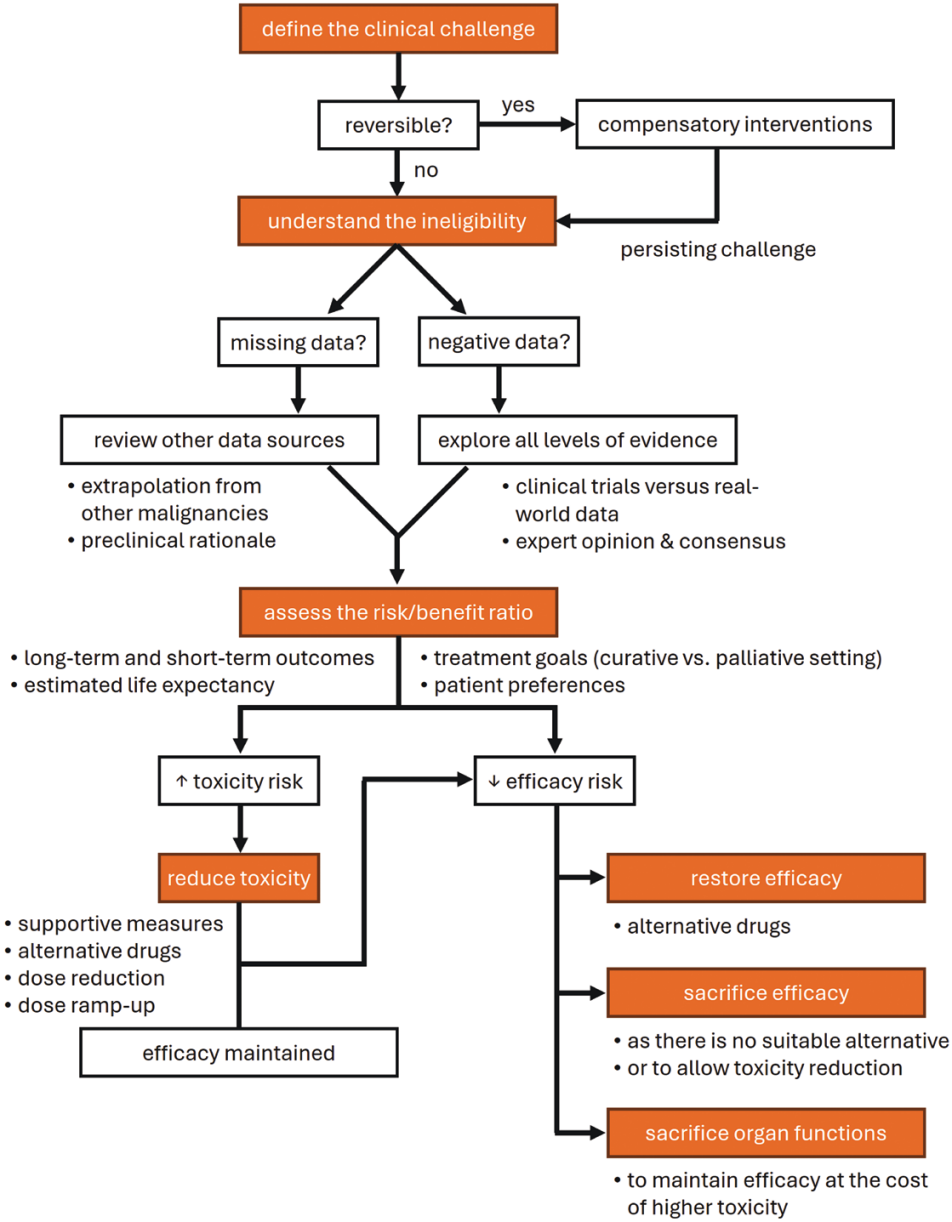
A practical guide to handle patients with challenging factors. Examples of clinical situations that may potentially lead to an increased risk of toxicity and decreased efficacy are listed in Tables 2 and 3, respectively.

As depicted in Figure 3, the process has 4 principal phases, starting with the definition of the clinical challenge (see also Table 1), followed by understanding the reasons for ineligibility, that is, contraindication for a given therapy, then assessing the risk to benefit ratio, and concluding by taking action, which may comprise procedures to reverse the unwanted outcomes. Notably, some challenging patient factors may be reversible in a reasonable time, including blood disorders requiring transfusions, growth factor support, or vitamin supplementation. Other examples include the elimination of drug interactions with chronic medication, the introduction of new medicines to compensate for some chronic disorders, or nutritional support. In a systematic review, prehabilitation interventions in HNC patients encompassing nutrition, psychoeducation, and exercise had a favorable impact on various domains, improving quality of life, morbidity, and mortality.^53^

Ineligibility for certain therapy arises when data relating to the given issue (clinical challenge) is missing or insufficient. This does not necessarily preclude treatment delivery, but a rationale should be provided, which can be based on extrapolation of data relevant to other malignancies or on preclinical findings. Another reason for ineligibility is the existence of negative outcome data indicating that an approach should not be used in the presence of certain patient- or disease-characteristics, as mentioned above. In such cases, a comprehensive review at all levels of available evidence is needed, along with a search for confirmatory data reproduced by independent research groups.

The next step is to ascertain the risks and benefits of therapy. This phase is not straightforward and depends on the clinical setting, either curative in locoregionally advanced disease or palliative in a disease that is metastatic or refractory to local ablative methods, keeping in mind that a minority of patients with recurrent and/or metastatic disease may experience long disease-free survival, which, if extending beyond 5 years, could be biologically compatible with cure. Furthermore, patients’ preferences and their perception of life priorities and quality of life must be acknowledged. In the curative setting, a more aggressive approach may be chosen to increase the odds of cure despite increasing toxicity. In the palliative setting, quality of life, defined by treatment toxicity and the complexity of the treatment schedule, can be prioritized over efficacy. Short-term outcomes should be weighed against long-term outcomes with regard to the patient’s estimated life expectancy.

Finally, action must be taken to confront the risk of increased toxicity (Table 2) or decreased efficacy (Table 3) using one of the following 4 options, that is, reducing toxicity, restoring efficacy, sacrificing efficacy, or sacrificing organ functions. Toxicity can be reduced by introducing supportive care measures (eg, granulocyte growth factors), selecting alternative drugs with comparable efficacy (eg, carboplatin with 5-fluorouracil instead of cisplatin), reducing the peak doses of chemotherapy while maintaining cumulative dose exposure (eg, weekly instead of 3-weekly regimens), or progressively escalating the dose after treatment initiation (dose ramp-up). Efficacy is typically restored by selecting equipotent alternative treatments, if available, or maintaining adequate cumulative doses, as illustrated above by the example of weekly and 3-weekly regimens. In specific clinical scenarios, such actions may also involve compromising on efficacy or accepting certain toxicity issues. If efficacy is sacrificed, patients receive treatment that may be less effective due to its lower anticancer potency or necessary schedule adjustments aimed at avoiding excessive toxicity. Here, no therapeutic alternative with better outcomes is considered suitable, and these patients are typically treated with palliative intent. If organ function is sacrificed, patients may still receive an optimal therapeutic approach in terms of efficacy, but this is offsetby a higher risk of typically permanent toxicity. For example, patients treated with curative intent may be subjected to cisplatin despite pre-existing hearing loss if no similarly effective regimen is feasible.

### Question 7: How to manage patients with substance use disorders?

In squamous cell carcinoma of the head and neck unrelated to human papillomavirus (HPV) infection, the major risk factors are alcohol consumption and exposure to tobacco, which may take different forms including traditional smoking (cigarettes, cigars, cigarillos, pipes), alternative smoking (waterpipe, bidis, kreteks), reverse smoking, smokeless tobacco consumption (chewing, snuff, snus, dissolvable), and passive smoking. Other substance users, including those taking cannabis, opioids, stimulants, and hallucinogens, may have poor oral hygiene and nutrition and engage in higher-risk sexual behaviors, associated with the likelihood of contracting high-risk HPV, all of which amplify the probability of HNC development. A common observation of different substance use disorders is an increased prevalence of associated comorbidities, including respiratory, cardiovascular, gastrointestinal and liver diseases, cancers, reproductive health issues, neurological and psychiatric disorders, and infectious diseases.^54-59^

These patients therefore represent complex cases for decision-making and are at higher risk of increased toxicity due to comorbidities, as well as decreased efficacy due to non-compliance. Special attention should be paid to cardiovascular and pulmonary disorders, which may require specific baseline examinations, for example, echocardiography or pulmonary function tests. Similar precautions should be taken with respect to infectious diseases (human immunodeficiency virus, viral hepatitis), concomitant chronic medication, and patient compliance. In the curative setting, patients receiving radiotherapy with or without chemotherapy may be better managed in the hospital to avoid treatment interruptions that may occur in the outpatient setting owing to impaired treatment adherence. In the palliative setting, it may be necessary to opt for low-toxicity regimens with predictable adverse events, such as carboplatin and paclitaxel, where a sudden and temporary loss to follow-up does not usually lead to severe complications. In this respect, the administration of immunotherapy may represent a medical dilemma, particularly if given as monotherapy, because of the risk of early progression, hyperprogression, and immune-mediated toxicity, which may have serious consequences if not dealt with in a timely manner.

## Conclusions

In managing HNC patients with challenging factors, data interpretation is crucial and should take into account all available sources of evidence, ranked according to different levels of quality and possible bias. Certain clinical situations may necessitate prioritizing lower-level evidence based on expert panels and consensus statements, which may provide guidance on unclear or ambiguous areas, but rigorous conduct and careful selection of experts are essential. We have entered a new era where the growing impact of artificial intelligence is expected to enhance the synthesis and interpretation of research findings.^60^ Enrolment of patients with comorbidities and other challenging characteristics in late-phase clinical trials, including post-registration phase IV studies, is strongly encouraged to understand their outcomes, both in terms of efficacy and toxicity.
